# The role of long noncoding RNAs in hepatocellular carcinoma

**DOI:** 10.1186/s12943-020-01188-4

**Published:** 2020-04-15

**Authors:** Zhao Huang, Jian-Kang Zhou, Yong Peng, Weifeng He, Canhua Huang

**Affiliations:** 1 State Key Laboratory of Biotherapy and Cancer Center, West China Hospital, and West China School of Basic Medical Sciences & Forensic Medicine, Sichuan University, and Collaborative Innovation Center for Biotherapy, Chengdu, 610041 China; 2 Institute of Burn Research, Southwest Hospital; State Key Laboratory of Trauma, Burn and Combined Injury, Chongqing Key Laboratory for Disease Proteomics, Army Military Medical University, Chongqing, 400038 China

**Keywords:** Long noncoding RNA, Hepatocellular carcinoma, Biomarker, Targeted therapy

## Abstract

Hepatocellular carcinoma (HCC) is the most frequent subtype of primary liver cancer and one of the leading causes of cancer-related death worldwide. However, the molecular mechanisms underlying HCC pathogenesis have not been fully understood. Emerging evidences have recently suggested the crucial role of long noncoding RNAs (lncRNAs) in the tumorigenesis and progression of HCC. Various HCC-related lncRNAs have been shown to possess aberrant expression and participate in cancerous phenotypes (e.g. persistent proliferation, evading apoptosis, accelerated vessel formation and gain of invasive capability) through their binding with DNA, RNA or proteins, or encoding small peptides. Thus, a deeper understanding of lncRNA dysregulation would provide new insights into HCC pathogenesis and novel tools for the early diagnosis and treatment of HCC. In this review, we summarize the dysregulation of lncRNAs expression in HCC and their tumor suppressive or oncogenic roles during HCC tumorigenesis. Moreover, we discuss the diagnostic and therapeutic potentials of lncRNAs in HCC.

## Introduction

Hepatocellular carcinoma (HCC) is one of the most common malignancies worldwide and ranks as the third most common cause of cancer-related death, especially in Africa and Eastern Asia due to the lack of surveillance and treatment options [1]. The risk factors underlying HCC pathogenesis are highly variable, including chronic hepatitis B (HBV) and hepatitis C virus (HCV) infection, alcohol consumption, non-alcoholic fatty liver disease (NAFLD), and aflatoxin B1 intake [2]. These factors may induce DNA damage, epigenetic alterations and cancer-related mutations, leading to the silencing of tumor suppressors (e.g. TP53, CDH1, RASSF1) and the activation of oncogenes (e.g. MYC, VEGFA, MAPK7), which eventually contribute to HCC progression [3–6]. To date, several preventive and therapeutic strategies have been implicated in the management of HCC, as exampled by the administration of anti-hepatitis vaccine, specific kinase inhibitors (e.g. Sorafenib and Regorafenib), surgical resection and liver transplantation [7–10]. These treatments, together with biomarker screening (e.g. a-fetoprotein), have minimized HCC-related death to a certain extent, but their performance is far from acceptable therapeutic effect, thus novel diagnostic/therapeutic approaches are still urgently needed to improve the clinic outcomes of HCC.

Early HCC-related studies mainly focused on the protein-coding genes due to their central roles in the regulation of biological processes. However, less than 2% of genome DNA encodes proteins, whereas the remaining 98% noncoding sequences and their RNA transcripts (ncRNAs) have been functionally uncharacterized and thought to be “noise” in the past [11]. Recently, increasing evidences indicate that these evolutionarily conserved ncRNAs, such as microRNA (miRNA), circular RNA (circRNA) and PIWI-interacting RNA (piRNA), particularly long noncoding RNA (lncRNA), play an important role in diverse physiological and pathological processes. In 1990, the first lncRNA H19, which restricts organ growth via decreasing IGF2 expression, was identified in fetal liver tissue [12, 13]. Next year, the lncRNA XIST mediating X chromosome inactivation was found [14]. To date, over 50,000 genes have been found to transcribe lncRNAs and their number is still rapidly growing [15]. These lncRNAs are differentially expressed in different tissues and cancers, including HCC [16]. Currently, the roles of most lncRNAs in HCC remain elusive, while a small part of which has been extensively investigated. For instance, lncRNA HULC is upregulated in HCC and has been shown to promote HCC growth, metastasis and drug resistance [17–19]. Intriguingly, some HCC-related lncRNAs are present in body fluids, which are easy to detect and analyze, making them have the potential to be attractive biomarkers in liquid biopsy of HCC. For instance, lncRNA-WRAP53 in serum is an independent prognostic marker to predict high relapse rate of HCC patients [20]. These vital roles and unique properties of lncRNAs suggest their potential clinical value in the HCC diagnosis and treatment. In this review, we briefly introduce dysregulation of lncRNAs expression in HCC, and summarize how lncRNAs regulate HCC progression by acting as tumor suppressor or oncogene. In addition, we discuss the diagnostic and therapeutic potential of lncRNAs in HCC.

## LncRNA as a modulator of liver microenvironment and chronic liver diseases

**Fig. 1:**
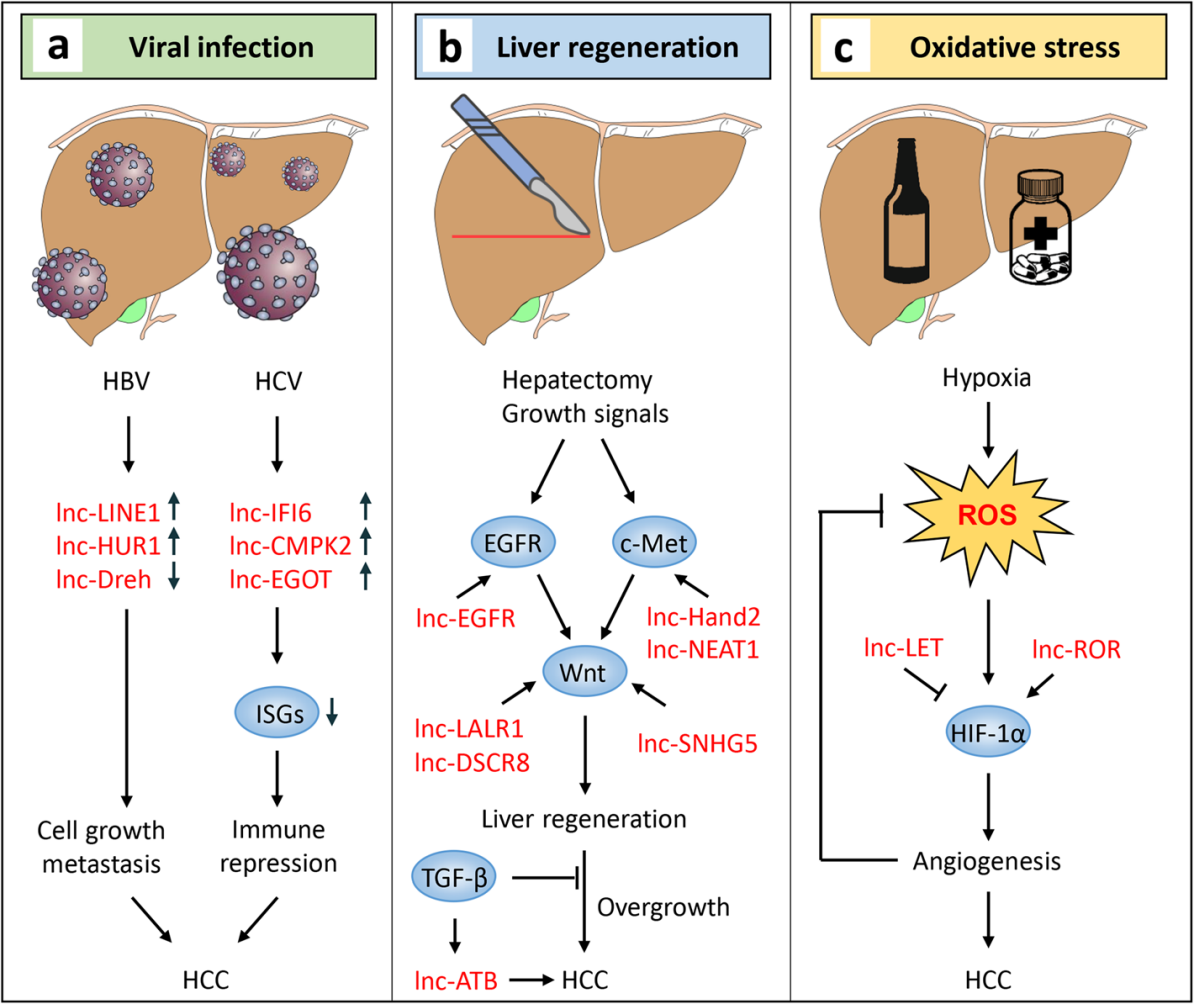
LncRNAs modulate liver microenvironment and chronic liver diseases. LncRNAs are involved in physiological and pathological processes, including (**a**) Viral infection and immune repression. Virus-induced lncRNAs are capable of promoting cell proliferation or suppressing host immune response, resulting in chronic infection and HCC formation. **b** Liver regeneration and outgrowth. In response to hepatectomy, liver is regenerated via activating proliferative signalings. These signalings can also be stimulated by certain lncRNAs independent of tissue loss, leading to liver overgrowth and HCC. **c** Hypoxic liver injury and oxidative stress. Drug detoxification induces ROS accumulation in liver, which upregulates HIF-1α to facilitate blood vessel formation therefore relief oxidative stress. To some similarities, several lncRNAs also activate HIF-1α to support HCC survival under hypoxic condition. ISGs, IFN-stimulated genes. ROS, reactive oxygen species

Liver cancer is distinguished from other cancer types by several unique features, including viral infection, regenerative signals and hypoxic microenvironment. These factors provide both opportunities and challenges for HCC cell proliferation and survival. Importantly, lncRNA modulates immune response, liver regeneration and redox signaling, which play critical roles in the regulation of liver microenvironment and chronic liver diseases. Dysregulation of lncRNA in these processes leads to chronic hepatitis, liver outgrowth and oxidative stress, which eventually result in the initiation and progression of HCC (Fig. 1).

### Viral infection

Hepatitis B virus (HBV) and hepatitis C virus (HCV) are two major viruses invading liver and causing chronic inflammation. More than 240 and 70 million people are infected respectively with HBV and HCV worldwide, a part of who tend to develop HCC if untreated [21, 22]. Hepatitis viruses hijack host cells to facilitate their replication or production via multiple mechanisms, one of which is regulating the transcription of certain lncRNAs. For instance, the HBV X protein (HBx) upregulates oncogenic lncRNA HUR1, which binds and blocks p53 to promote HCC growth [23]. Besides, HBx can also downregulate tumor suppressive lncRNA Dreh, leading to re-expression of vimentin and promoting HCC metastasis [24]. During infection, the host immune is activated to restrict viral replication. However, the invaded hepatitis viruses are able to alter Toll-like receptor (TLR) signaling, resulting in a defective immune response that is inadequate for virus clearance. It allows the virus to escape from immune surveillance, in which lncRNAs play crucial roles. It has been shown that infection of HCV upregulates the level of a series of lncRNAs such as IFI6, CMPK2 and EGOT that inhibit the expression of IFN-stimulated genes (ISGs), leading to immune suppression and chronic inflammation [25–27]. These findings indicate that viruses modulate antiviral response through lncRNAs encoded by host or itself genome. Intriguingly, lncRNA can also be generated from an integrated genome. Lau et al. reported a viral-human gene fusion that produces a chimeric lncRNA HBx-LINE1, which promotes HCC through activating Wnt signaling [28]. This chimeric lncRNA can be detected in 23.3% HBV bearing HCC samples and decrease the survival of HCC patients, suggesting HBV/HCV associated lncRNAs might be potential biomarkers in HCC risk evaluation and diagnosis.

### Liver regeneration

Liver is the only visceral organ capable of regeneration after partial hepatectomy or chemical injury. After a hepatectomy, hepatic growth factor (HGF) is released due to elevated urokinase activity, which potently activates EGFR and c-Met [29, 30]. EGFR and c-Met promote hepatocyte proliferation through activating multiple signalings, such as Wnt [31, 32]. Once regeneration is completed, TGF-β represses the function of urokinase and HGF thus brings liver cells back to the quiescent state [33]. Several lncRNAs are involved in this process. For example, lncRNA-LALR1 has been found to suppress Axin1 thus activating Wnt signaling, which stimulates hepatocyte proliferation during liver regeneration [34]. Besides, lncHand2 is capable of upregulating the expression of Nkx1–2, thereby activating c-Met in hepatocyte to facilitate liver regeneration [35]. However, persistent proliferative signaling induced by lncRNA dysregulation frequently leads to liver tumorigenesis. For instance, lncRNA DSCR8 [36] and SNHG5 [37] have been shown to upregulate Wnt and result in liver tumor growth, whereas lnc-EGFR [38] and lncRNA NEAT1 [39] are capable of activating EGFR and c-Met respectively, leading to HCC development. These lncRNAs are probably activated in the absence of liver tissue loss or failed to be silenced by quiescent signal TGF-β. Indeed, TGF-β may upregulate certain lncRNAs such as ATB to promote HCC metastasis [40], and the key regulatory mechanisms to determine whether an oncogenic or tumor suppressive role of TGF-β in this context remains unclear.

### Oxidative stress

Liver accounts for approximately 2% of body weight but receives about 25% of cardiac output. Sufficient blood supply is indispensable for its oxygen demand, whereas poor oxygen delivery or inadequate antioxidant machinery frequently results in hypoxic liver injury, an oxidative stress-associated liver disease [41]. In addition, various risk factors such as alcohol consumption, viral infection, drug detoxification and metabolism also markedly induce accumulation of reactive oxygen species (ROS), leading to oxidative stress in liver. To adapt to this hypoxic microenvironment, HCC cells develop survival strategies such as promoting angiogenesis, thereby receiving additional oxygen supply from the bloodstream [42]. This process involves HIF-1α, a master transcription factor that facilitates angiogenesis. Recent studies revealed that several lncRNAs are sensitive to hypoxia and might function through HIF-1α. One of hypoxia-responsive lncRNAs is linc-RoR, which is induced by oxidative stress and upregulates HIF-1α to support HCC cell survival [43]. In contrast, lncRNAs that negatively regulate HIF-1α, including lncRNA LET, are downregulated during HCC progression [44]. Mechanistically, lncRNA-LET interacts with a RNA-binding protein NF90 and promotes its degradation, leading to destabilization of HIF-1α mRNA. In response to hypoxia, HDAC3 is induced and represses the transcription of lncRNA-LET, resulting in expression of HIF-1α and HCC metastasis [44]. For protecting liver from oxidative damage, natural antioxidant supplies including green tea, jujube honey and virgin olive oil are encouraged [45], but the roles of lncRNAs in this process is yet to be determined.

## Aberrant expression of lncRNA in HCC

The biogenesis of lncRNAs shows high similarities with that of protein-coding transcripts. Epigenetic modification, transcription complex recruitment and RNA processing are major events regulating lncRNA biogenesis. Before transcription, active chromatin marks are commonly present within lncRNA promoter, including the acetylation of H3K9 and H3K27, as well as the dimethylation or trimethylation of H3K4 [46]. In contrast, the trimethylation of H3K27 is recognized as a repressive chromatin mark. For example, knockdown of EZH2, a methyltransferase responsible for H3K27me3 modification, has been shown to reactivate the transcription of lncRNAs in embryonic stem cells [47]. These evidences indicate that the epigenetic modification of lncRNA genes may follow nearly the same regulatory rules as protein-coding genes. Interestingly, another transcriptional repression mark H3K9me3 is more enriched in the promoter region of lncRNAs compared with mRNAs, suggesting some unique features of lncRNAs in their histone modification patterns [48]. The detailed mechanisms underlying these epigenetic modifications remain elusive, whereas some chromatin remodeling complex such as Isw2, Swr1, Ino80 and Rsc are probably involved [49].

Next, in the presence of active chromatin marks or the absence of repressive marks, RNA polymerase II is recruited to initiate the transcription of lncRNAs, sometimes in a bidirectional way to yield divergent transcripts. Generally, lncRNA genes tend to share the same promoter with their neighbor protein-coding genes [50]. Besides, some other transcription patterns have been extensively reviewed elsewhere [51]. Then, the majority of nascent transcripts are processed into mature lncRNAs through canonical ways like mRNAs, including methylguanosine capping at 5′ end, polyadenylation to form a poly-A tail at 3′ end, and splicing to remove introns. Alternatively, some lncRNAs have been shown to undergo non-canonical processing ways. For instance, the lncRNA MALAT1 and MENβ are cleaved by tRNA processing enzymes RNase P and RNase Z to form triple-helical structures at their 3′ end, which protects them from degradation and facilitates their cellular accumulation in the absence of poly(A) tail [52, 53]. In summary, the biogenesis of lncRNA and mRNA is broadly similar, but considerable differences exist and endow these RNA molecules with unique features and functions.

**Fig. 2:**
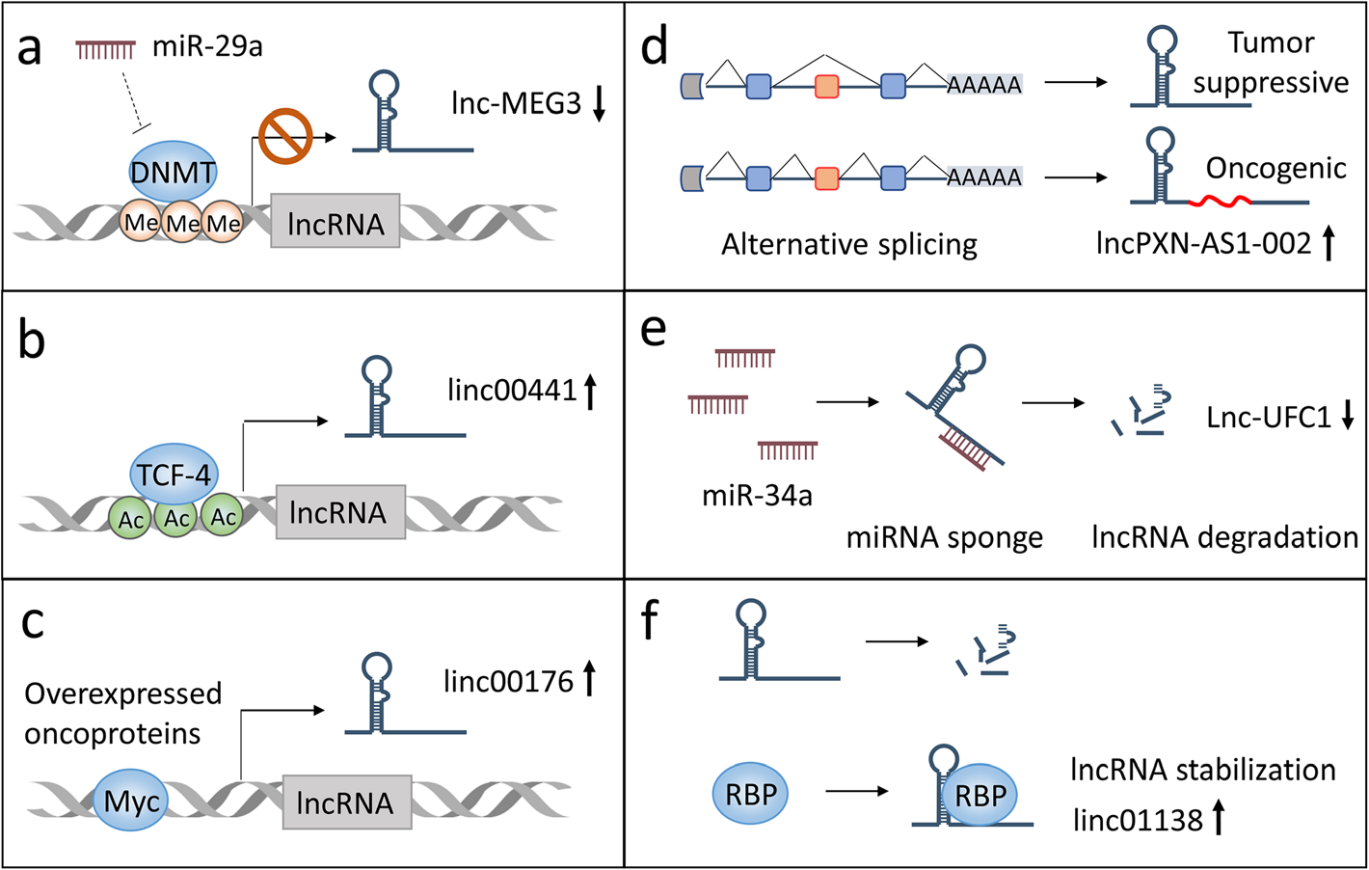
Abnormal expression of lncRNA in HCC. **a** Epigenetic silencing of HCC-suppressive lncRNAs; **b** Epigenetic activation of HCC-promoting lncRNAs; **c** Transcriptional activation by HCC-related oncoproteins; **d** RNA processing that endows lncRNA with HCC-promoting properties; **e** Loss of tumor-suppressive miRNAs leads to re-expression of targeted oncogenic lncRNAs; **f** RBP-induced stabilization of HCC-promoting lncRNAs. RBP, RNA-binding protein

Aberrant lncRNA biogenesis has been implicated in the pathogenesis of various diseases, including HCC. Indeed, high-throughput platforms such as RNA-sequencing and microarray have revealed distinct lncRNA expression profiles in HCC tissues compared with noncancerous liver tissues (Table 1), suggesting that biogenesis of certain lncRNAs is dysregulated during HCC development [54, 73]. These aberrant biogenesis events basically include the epigenetic silence/activation of tumor-suppressive/promoting lncRNAs, the transcriptional activation/repression of lncRNAs by certain oncogenic/tumor-suppressive transcription factors, the special processing patterns that endow lncRNAs with oncogenic functions, and the binding of lncRNAs with microRNA or proteins that affect lncRNA stability (Fig. 2).

**Table 1:** Some dysregulated LncRNAs and their roles in the progression of HCC

LncRNAs	Roles in HCC	Binding partners	Action modes	Outcomes	Refs
Dreh	Tumor suppressor	Vimentin protein	Cytoskeleton reorganization	↓ metastasis ↓ proliferation	[24]
ATB	Oncogene	miR-200	miRNA sponge	↑ metastasis	[40]
UFC1	Oncogene	β-catenin mRNA HuR protein	mRNA stabilization	↑ proliferation	[54]
PXN-AS1	Oncogene	PXN mRNA	mRNA stabilization	↑ proliferation	[55]
ANRIL	Oncogene	EZH2 protein Target gene DNA	Transcriptional suppression	↑ proliferation	[56]
TUG1	Oncogene	EZH2 protein Target gene DNA	Transcriptional suppression	↑ proliferation ↑ glycolysis	[57, 58]
lnc-β-Catm	Oncogene	EZH2 protein β-catenin protein	Protein stabilization	↑ stemness	[59]
GIHCG	Oncogene	EZH2 protein Target gene DNA	Transcriptional suppression	↑ proliferation ↑ metastasis	[60]
DANCR	Oncogene	β-catenin mRNA	mRNA stabilization	↑ stemness	[61]
MALAT1	Oncogene	miR-143-3p	miRNA sponge	↑ metastasis ↑ proliferation	[62]
HULC	Oncogene	miR-186 miR-9	miRNA sponge	↑ proliferation ↑ lipogenesis	[63, 64]
XIST	Tumor suppressor	miR-92b	miRNA sponge	↓ metastasis ↓ proliferation	[65]
PVT1	Oncogene	NOP2 protein	Protein stabilization	↑ stemness	[66]
HOTTIP	Oncogene	miR-192 miR-204	miRNA sponge	↑ glutaminolysis	[67]
DILC	Tumor suppressor	IL-6 DNA	Transcriptional suppression	↓ stemness	[68]
ICR	Oncogene	ICAM-1 mRNA	mRNA stabilization	↑ stemness	[69]
ZFAS1	Oncogene	miR-150	miRNA sponge	↑ metastasis	[70]
MVIH	Oncogene	PGK1 protein	Protein localization	↑ metastasis	[71]
CASC9	Oncogene	HNRNPL protein	Protein phosphorylation	↑ proliferation	[72]

### Epigenetic modification

During HCC progression, abnormal chromatin marks including DNA methylation and histone modification at lncRNA genes are universally identified [74], leading to the decrease of HCC-repressive lncRNAs (Fig. 2a) or increase of HCC-promoting lncRNAs (Fig. 2b). For instance, DNA methyltransferases DNMT1 and DNMT3 induce hypermethylation at the promoter of tumor-suppressive lncRNA MEG3, whose epigenetic silence results in apoptosis resistance of HCC cells and liver tumor growth [75]. In contrast, some HCC-promoting lncRNAs are induced by activated epigenetic marks. Tang et al reported that the upregulation of linc00441 expression was due to enhanced H3K27 acetylation [76]. Overexpressed linc00441 in turn recruits DNMT3A to methylate and inactivate its neighbor gene RB1, thereby promoting the proliferation of HCC cells [76].

### Transcriptional activation

It has been well established that a series of oncogenic transcriptional factors/co-factors are overexpressed in HCC, including YAP, c-Myc and β-catenin. These transcriptional factors initiate the transcription of not only protein-coding genes but also non-coding genes such as lncRNAs. For example, a systemic study revealed that the transcription of numerous lncRNAs is regulated by the transcription factor Myc [77] (Fig. 2c). Given that enhanced Myc signaling in HCC, a class of lncRNAs should be at least partially responsible for Myc-driven HCC progression. Indeed, it has been reported that lncRNA linc00176, which overexpresses exclusively in HCC, is transcribed by Myc. Depletion of this Myc-regulated lncRNA led to cell cycle arrest and necroptosis in HCC cells, suggesting that lncRNA transcription contributes largely to the oncogenic function of HCC relevant oncoproteins [78].

### RNA processing

Certain primary lncRNA transcripts undergo special processing, such as exon inclusion (Fig. 2d). These processing modes might provide oncogenic function for HCC development. A splicing factor MBNL3, which is overexpressed in fetal liver and HCC tissue but loss in adult normal liver, has been shown to induce exon 4 inclusion of lncRNA PXN-AS1. This alternative splicing event allows lncRNA PXN-AS1 to interact with the PXN mRNA and protects it from degradation, leading to its overexpression and consequent HCC development [55].

### MiRNA sponging

Several lncRNAs function as competitive endogenous RNA (ceRNA) to bind miRNAs therefore also known as miRNA sponge. This lncRNA-miRNA association leads to the re-expression of miRNA target genes, which will be discussed in the next section. Not surprisingly, sponging of miRNAs affects the expression of lncRNA itself, providing another layer of post-transcriptional regulation of lncRNAs (Fig. 2e). It has been shown that miR-34a directly binds lncRNA-UFC1 to decrease its half-life, thereby suppressing HCC cell proliferation induced by lncRNA-UFC1 [54]. Given that miRNAs are globally downregulated during HCC development, more oncogenic lncRNAs are expected to be reactivated thus form an aberrant lncRNA expression profile.

### Protein binding

Besides miRNAs, some kinds of proteins can also bind lncRNAs to regulate their turnover. These proteins mainly include RNA-binding proteins (RBPs), which are conventionally known as binding mRNA. In terms of considerable similarities existed between lncRNAs and mRNAs, many RBPs have been found to regulate lncRNA stability through physical interaction (Fig. 2f). Among these RBPs, insulin like growth factor-2 mRNA-binding proteins 1/3 (IGF2BP1/3) binds and stabilizes linc01138 on its 220–1560-nt fragment, which is required for the growth and metastasis of HCC cells [79]. Moreover, the aforementioned lncRNA UFC1 is able to interact with another RBP termed human antigen R (HuR) through its 1102–1613-nt fragment, which is indispensable for the HCC-promoting function of lncRNA-UFC1 [54]. These evidences suggest that abnormal lncRNA biogenesis in HCC can be attributed to RBP-regulated lncRNA decay.

## Functional mechanisms of lncRNA in HCC

**Fig. 3:**
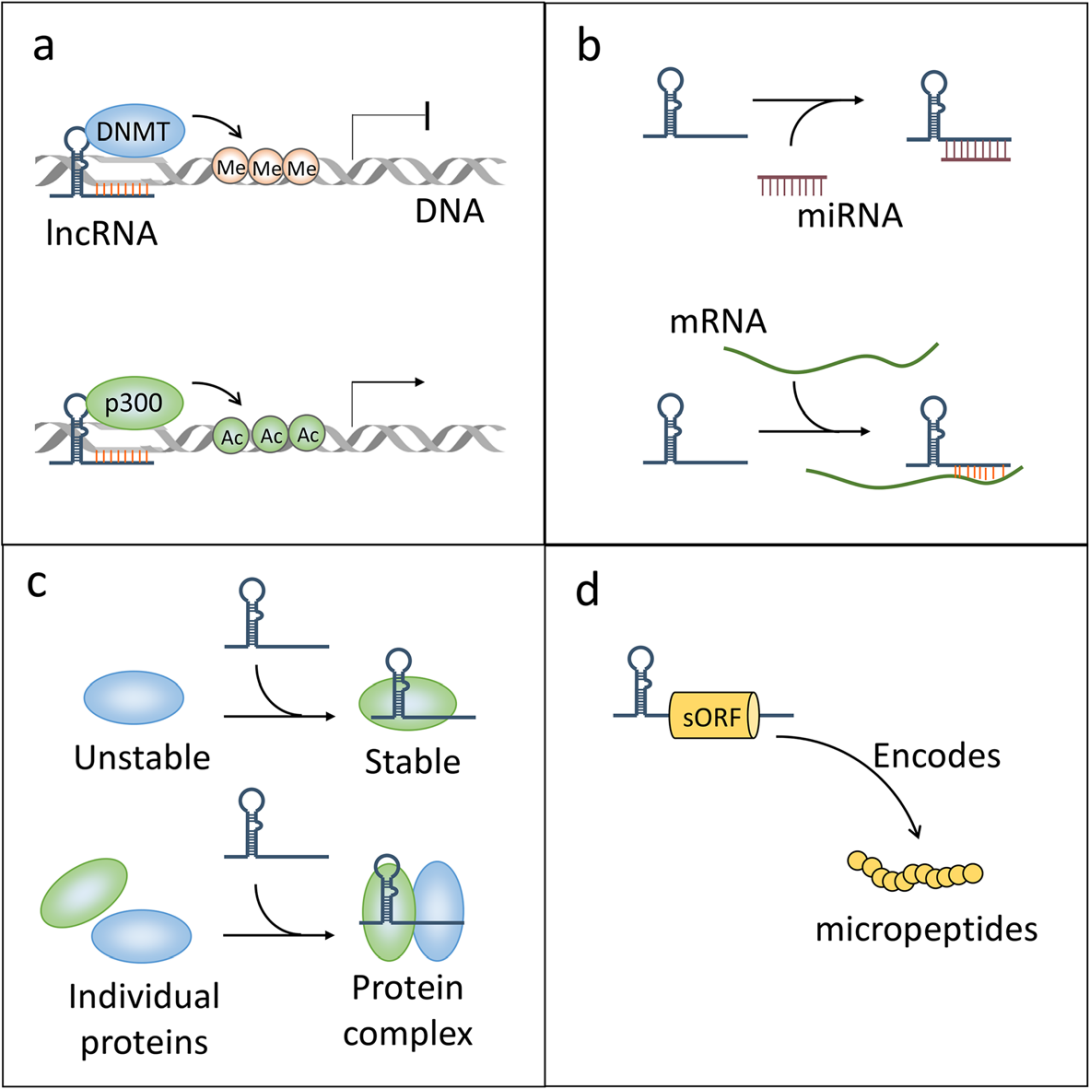
Functional mechanisms of lncRNA in HCC. **a** LncRNAs interacts with DNA to regulate gene expression through binding with promoters or distal regulatory elements, where they recruit epigenetic modifiers to promote or repress gene expression. **b** LncRNAs bind to mRNAs to regulate their stability, thereby regulating gene expression. Besides, lncRNAs can also bind to miRNAs as molecular sponge, leading to the reactivation of genes targeted by miRNAs. **c** LncRNAs interact with proteins to regulate their stability or facilitate the formation of protein complex when they function as scaffold. **d** Several lncRNAs hold the potential to encode small peptide, through which exhibit their biological functions

LncRNAs exert their functions to regulate cell fate through multiple ways, including DNA binding, RNA interaction, associating with proteins, and producing small peptides. Firstly, binding to DNA allows lncRNAs to remodel chromatin structure and epigenetic modifications, thus regulating the expression of target genes. Secondly, lncRNAs interact with mRNAs or miRNAs as molecular sponge, thereby modulating the stability and translation of mRNAs or the binding of miRNAs with their own targets. Thirdly, lncRNAs are capable of binding with proteins to regulate their conformation, localization or stability, the formation or disassociation of protein complex as well as other aspects of functions. In addition, a part of lncRNAs contain small open reading frames (sORFs) that encode peptides with biological functions. Compelling evidences suggest that lncRNAs participate in HCC progression through regulation of their binding partners and peptide-coding properties (Fig. 3).

### DNA binding and chromatin remodeling

Nuclear lncRNAs are widely observed to be associated with DNA, including protein-coding or non-coding regions (Fig. 3a). This RNA-DNA binding allows lncRNAs to regulate genes in *cis* (lncRNA genes and target genes are localized at the same chromosome) or in *trans* (different chromosomes). A well-characterized lncRNA that binds DNA is XIST, which mediates X chromosome inactivation during the early developmental process [14]. At present, a growing number of lncRNAs have been shown to bind with DNA, however, only a small fraction has been functionally annotated. For instance, a high-throughput study identified 276 lncRNAs interacting with DNA, among which lncRNA MEG3 was further validated to be capable of regulating the TGF-β pathway through interacting with DNA [80]. The typical lncRNA-DNA interaction site may lie in the promoters or other regulatory DNA sequences (such as enhancers) of certain genes, thereby regulating gene expression. For example, lncRNA TUG1 binds with the promoter region of PGC-1α gene, enhancing the promoter activity and resulting in PGC-1α overexpression [81]. Mechanistically, this transcriptional control by lncRNA-DNA interaction is usually achieved through the formation of RNA-DNA hybrid (also known as R loop) to alter chromatin structure [82]. These changed chromatin structure may provide signals to recruit biomolecules (such as epigenetic modifiers) to modulate gene expression, or spatially bring promoters and enhancers/repressors together to regulate the transcriptional activities. It has been reported that lncRNA-DNA hybrid has been found to accelerate transcriptional induction, which is implicated in the fast adaptation of organisms to environmental stimuli [83]. Another report has shown that lncRNA Khps1 directly interacts with the promoter of SPHK1 gene to form a lncRNA-DNA structure, where it recruits histone acetyltransferase p300/CBP to activate SPHK1 expression [84].

HCC can be in part attributed to particular lncRNA-DNA interaction. Abnormal chromatin marks are frequently observed during HCC development largely due to dysregulation of epigenetic modifiers, including DNMTs, EZH2, HDACs, PCAF, and other effectors that regulate these epigenetic modifying enzymes [85–87]. Importantly, accumulating evidences have suggested that some of these epigenetic modifiers can be regulated by lncRNAs in HCC, leading to aberrant epigenetic changes such as hypomethylation, hypermethylation and other modifications [74]. For instance, UHRF1 drives HCC through inducing global DNA hypomethylation, and further study revealed that lncRNA UPAT can directly bind to UHRF1 and therefore protect it from degradation, indicating that lncRNA UPAT-mediated UHRF1 stabilization may be an oncogenic factor of HCC [88, 89]. Besides, hypermethylation of certain genes are also involved in HCC progression. That situation includes the two lncRNAs ANRIL and TUG1, which are both able to recruit EZH2 to induce H3K27me3 modification at the promoter region of KLF2, thus repressing its transcription and inhibiting HCC cell growth [56, 57]. Interestingly, many other lncRNAs, such as HOTAIR, lnc-β-Catm, GIHCG and UCA1, have been also shown to be capable of recruiting EZH2 to play certain roles in the progression of HCC, suggesting an extensive connection between EZH2 and lncRNAs [59, 60, 90, 91]. Given that epigenetic modification is a reversible process that differs from gene mutation, targeting lncRNA-mediated epigenetic regulation might be a promising strategy for HCC treatment.

### Sponging mRNAs and miRNAs

RNR-RNA interaction represents a crucial mechanism to regulate cellular events through complementary base-pairing. It has been extensively investigated that miRNAs bind mRNAs to regulate mRNA turnover, thus affecting gene expression [92]. LncRNAs are capable of interacting with both mRNAs and miRNAs, making RNA regulation more comprehensive (Fig. 3b). Generally, lncRNA-mRNA interaction regulates the stability and localization of bound mRNAs, whereas lncRNA-miRNA interaction represses the binding of miRNAs with their own targets, namely miRNA sponge (also known as competitive endogenous RNA, ceRNA). For instance, a genome-scale RNA interactome study has shown a critical role of lncRNA TINCR in somatic tissue differentiation through interacting with a variety of differentiation-related mRNAs. Mechanistically, TINCR binds to those mRNAs harboring a 25-nt TINCR box motif, and then the STAU1 protein is recruited to interact with the TINCR-mRNA complex, therefore stabilizing the bound mRNAs and promoting their expression [93]. Another lncRNA MALAT1 has been shown to interact with nascent pre-mRNAs, thus localizing to active chromatin sites, which may facilitate the processing of pre-mRNAs [94]. Besides the regulation of mRNA stability and translocation, lncRNAs frequently function as molecular sponge to regulate numerous miRNAs through direct binding. Similar to mRNA binding, those lncRNAs associating with miRNAs usually contain specific binding region. One such example is lncRNA H19, which contains two functional binding sites of let-7 miRNA. Such binding releases let-7 from and activates the expression of its targets, Dicer and Hmga2 [95]. Another study using miR-CLIP approach identified H19 functions as miR-106a sponge and such interaction plays vital roles in the differentiation of skeletal muscle cells [96]. Interestingly, lncRNA-miRNA interaction sometimes affects cellular processes by modifying the function of lncRNA itself, but not the bound miRNA. For instance, LINC00673 acts as a tumor suppressor by inhibiting SRC-ERK signaling, whereas a G to A nucleotide mutation on it creates a binding site for miR-1231, which represses the antitumor function of LINC00673 [97].

The crosslink between lncRNAs and other RNA molecules (mRNAs and miRNAs) is an emerging issue related to HCC progression. Certain lncRNAs, such as UFC1, can stabilize β-catenin mRNA through binding with the mRNA stabilizing protein HuR, leading to the activation of Wnt signaling and consequent HCC progression [54]. Another lncRNA DANCR stabilizes the mRNA of β-catenin via direct binding, thus promoting HCC [61]. More generally, however, lncRNAs indirectly affect the stability of mRNAs through regulation of miRNAs, but not the direct lncRNA-mRNA binding. Indeed, miRNAs have been shown to globally downregulated during HCC development, which can be at least partially attributed to defects in miRNA processing machinery, the impaired pre-miRNA nuclear export, and the lncRNA-mediated miRNA dysregulation [98–100]. There are two major mechanisms underlying miRNA regulation by lncRNAs, namely epigenetic modification and physical association (miRNA sponge). Epigenetic regulation of miRNAs by lncRNAs including HOTAIR and GIHCG has been discussed above, and some epigenetic modifiers such as EZH2 are required for this process. In contrast, a large number of lncRNAs including MALAT1, FTX and MUF can directly bind to miRNAs to regulate their function [62, 101, 102]. These miRNA sponges interrupt the association between miRNAs and their target mRNAs to result in the reactivation of certain HCC-related genes. One of such cases is the well-known lncRNA HULC, which acts as a competing endogenous RNA for miR-186, thereby upregulating HMGA2 to support HCC growth [63]. Alternatively, some miRNA sponge such as lncRNA XIST functions as a tumor suppressor via interacting with miR-92b, which represses the proliferation and metastasis of HCC [65]. It is worth noted that a single lncRNA might bind a variety of RNA species simultaneously, suggesting the binding of lncRNAs with other RNA species can be effective and multi-functional.

### Protein interaction and regulation

The association of lncRNAs with proteins orchestrates protein localization or stability, as well as protein complex assembly or sequestration of proteins from their own binding partners, thereby executing their biological function (Fig. 3c). For instance, lncRNA XIST plays a central role in the X chromosome inactivation, and this function is closely related to the control of protein localization. Chen et al. recently reported that XIST binds with the LBR thereby directing the inactivated X chromosome to localize at the nuclear lamina, where XIST can spread across the X chromosome to silence transcription [103]. Besides, various lncRNAs, such as FAL1, AB074169 and MALAT1, have been shown to regulate the stability of bound proteins including BMI1, KHSRP and SREBP-1c, respectively [104–106]. This may be due to an altered binding affinity between protein turnover machinery and lncRNA-bound proteins. In addition to regulating protein localization and stability, lncRNAs can also mediate assembly of protein-protein complex via function as a scaffold. For example, two ends of lncRNA HOTAIR bind respectively with PRC2 and LSD1, thus tether these two distinct histone modifiers together to form a new protein complex [107]. Moreover, a recent study reported that lncKdm2b interacts with SRCAP protein to facilitate the assembly of SRCAP remodeling complex, promoting the renewal of embryonic stem cells [108]. In contrast, some lncRNAs act as competitive binding partners to sequester proteins from their original substrates, leading to the disassociation of protein complex. Such lncRNAs include Mhrt, which competitively interacts with the helicase domain of Brg1, leading to the disassociation of Brg1 and its genomic DNA targets thereby repressing Brg1-induced chromatin remodeling [109]. Similarly, lncRNA NORAD has been shown to sequester PUMILIO proteins from mRNAs, which is implicated in the maintenance of genomic stability [110].

Numerous studies have indicated that lncRNA-protein interaction play key roles in the pathogenesis of HCC through distinct mechanisms, including regulation of protein localization and stability, as well as complex assembly and protein sequestration. 1) The subcellular localization of many HCC-related proteins has been reported to be regulated by lncRNAs. For instance, lncRNA MUF interacts with AXNA2 to promote its cytoplasmic translocation, where AXNA2 activates WNT signaling thus leading to HCC progression [102]. 2) LncRNA-mediated stabilization of oncoproteins and tumor suppressors can result in the progression and suppression of HCC, respectively. One of such oncoprotein NOP2 can be bound and stabilized by lncRNA PVT1, therefore promoting the proliferation of HCC cells [66]. In contrast, the well-known tumor suppressor p53 is ubiquitinated and degraded by MDM2, which has been fully documented. LncRNA PRAL facilitates the interaction of p53 with HSP90 thus preventing the binding of p53 with MDM2, leading to the stabilization of p53 and the suppression of HCC growth [73]. Similarly, lncRNA PSTAR physically binds to hnRNP K protein and enhances its SUMOylation, which facilitates the formation of the p53-hnRNP K protein complex, thereby competitively blocking MDM2-dependent p53 protein degradation and inhibiting HCC cell proliferation [111]. 3) LncRNAs HOTAIR is capable of serving as a scaffold to bind with PRC2 and E3 ligase Mex3b simultaneously, facilitating the degradation of PRC2 and promoting HBV-induced HCC progression [112]. 4) LncRNAs can also sequester proteins from their substrates to play some roles in HCC. For example, DNA replication licensing factor MCM2 promotes DNA replication and is related to HCC cell division. LncRNA FTX binds with MCM2 thereby sequestering it from chromatin, leading to cell cycle arrest and inhibition of HCC growth [101]. Unlike interacting with RNA species via base-pairing, lncRNAs bind proteins mainly through their complex secondary structure. Though several tools have been developed to predict the structure of lncRNAs [113], the mechanism underlying lncRNA-protein interaction is yet to be elucidated due to lack of in-depth researches.

### Encoding small peptides

LncRNAs are defined as a kind of long transcripts that do not encode proteins. However, recent studies revealed that some lncRNAs indeed contain small open reading frames (sORFs, less than 100 amino acids) producing functional peptides (Fig. 3d). As early as 2002, a plant-derived lncRNA ENOD40 was found to encode two small peptides (12 and 24 aa) that interact with sucrose synthase [114]. Following study indicated that the 12-aa peptide binds sucrose synthase through disulfide bond, elevating the sucrose cleavage activity of sucrose synthase [115]. With the development of bioinformatic and sequencing technologies in recent years, more sORFs have been identified in transcripts that previously annotated as non-coding. For instance, ribosome profiling and Poly-Ribo-Seq are able to screen ribosome-associated RNAs, many of which are lncRNAs thus suggest their coding potential [116, 117]. Further analysis revealed that 40% of human lncRNAs might be translated, especially those located in the cytoplasm [118]. However, it is worth noted that binding to ribosome does not mean that they are truly translated. To validate the production of peptides, a mass spectrometry-based approach has been applied to confirm that only a small subset of lncRNAs are translated, which may be due to the rapid degradation of small peptides [119].

To date, very few efforts have been devoted to elucidating the functions of lncRNA-encoded small peptides in HCC development. Recently, a relevant study using the ribosome profiling method revealed that a 99-aa peptide termed KRASIM, which is encoded by lncRNA NCBP2-AS2, binds KRAS protein to inhibit ERK signaling, leading to the suppression of HCC cell growth [120]. The lack of relevant study might be attributed to multiple reasons. Firstly, the small peptides are probably unstable therefore not easy to detect. Secondly, a fraction of these small peptides are not homolog to well-annotated proteins, making it difficult to predict their function. It is also possible that a subset of micropeptides actually have no biological function. Therefore, further investigations are required to better characterize the roles of lncRNA-encoded peptides in HCC.

## Significance of lncRNA in HCC hallmarks

**Fig. 4:**
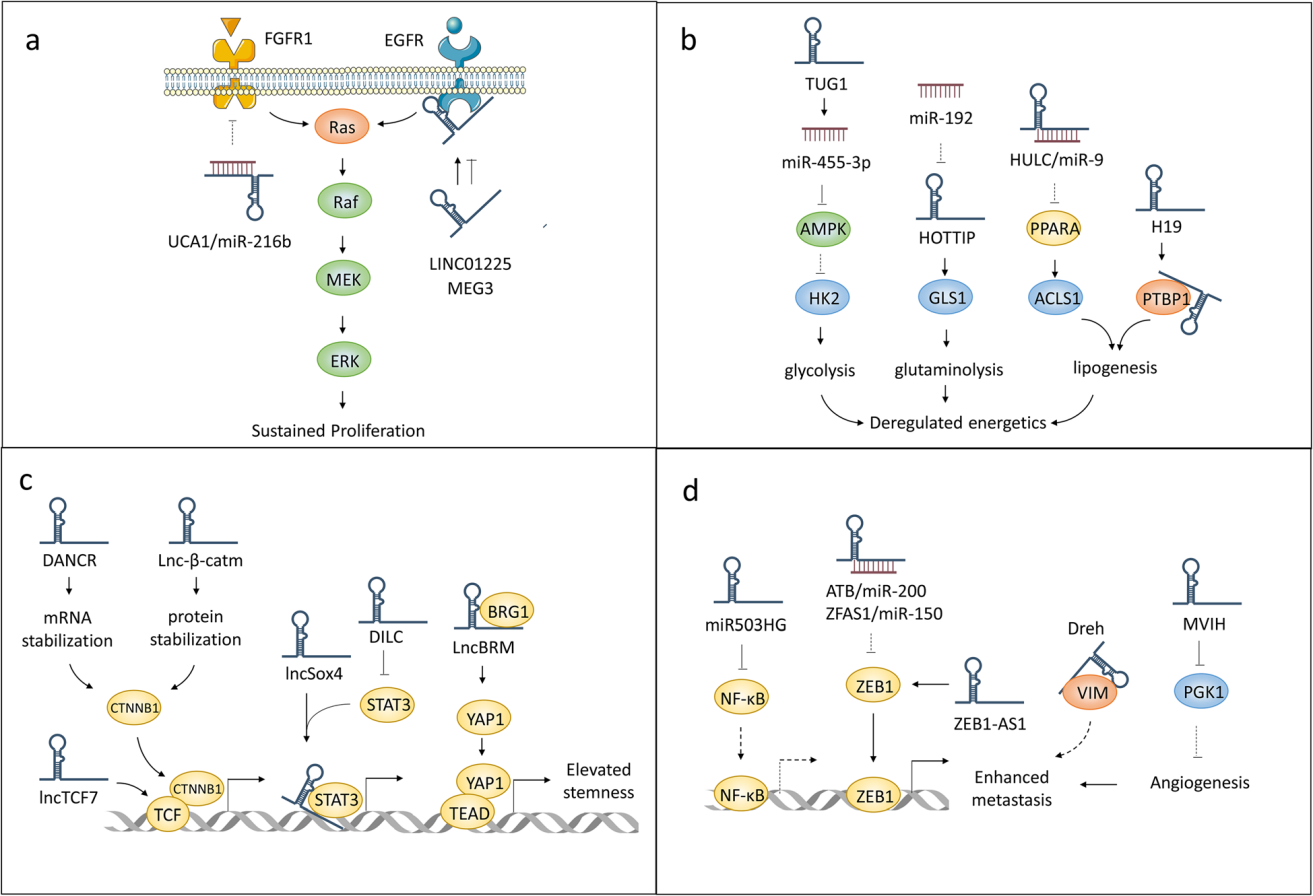
Significance of lncRNA in HCC hallmarks. Dysregulated lncRNAs promote HCC through distinct mechanisms, leading to different phenotypes corresponding to every tumor stage. These hallmarks include (**a**) sustained proliferation due to aberrant regulation of growth factor receptor/RTK signaling or cell cycle progression; **b** dysregulated energetics induced by metabolic reprogramming; **c** elevated stemness due to the hyperactivation of CSC-related signaling; **d** enhanced metastasis induced by the overexpression of mesenchymal markers and formation of additional vessels

Similar hallmarks are shared by different cancer types, including persistent proliferation, resistant to cell death, and elevated invasive capability [121]. HCC-related lncRNAs promote or suppress these cancerous phenotypes through the interplay with their binding partners, underlying rational therapeutic strategies for the treatment of HCC (Fig. 4).

### Sustaining proliferative signaling

Cell proliferation is basically triggered by receptor tyrosine kinases (RTKs) activation and cell cycle progression. Normal cells restrict their proliferation to maintain a proper architecture of organisms, while constitutive activation of RTKs or bypass of cell cycle checkpoints can lead to tumorigenesis, including HCC. On one hand, hyperactivation of RTKs such as EGFR, c-Met, IGF1R and IGF2R, have been reported to play important roles in supporting HCC growth [122, 123]. This may largely due to constitutive activation of their downstream signaling including PI3K/Akt and Ras/Raf/MEK/ERK pathways, resulting in a high proliferation rate of HCC cells. On the other hand, the progression of cell cycle is also required for cell proliferation. Generally, cell cycle is precisely regulated by cyclins and cyclin-dependent kinases, which provide the permission of several checkpoints such as G1/S and G2/M. Dysregulation of checkpoint proteins including Cyclin D1, CDK4 and CDK6 have been positively correlated with HCC cell proliferation [124–126].

Not surprisingly, some lncRNAs are involved in the HCC progression by regulating such RTKs and cell cycle (Fig. 4a). For example, lnc-EGFR interacts with EGFR to lead to its constitutive activation, which promotes HCC growth [38]. EGFR can also be bound by LINC01225, therefore increasing its protein level, contributing to the activation of EGFR-Ras signaling and sustaining HCC cell proliferation [127]. Besides EGFR, other HCC-related RTKs such as c-Met, IGF1R and IGF2R have been also shown to be regulated by lncRNAs, suggesting a wide range of lncRNA-RTK regulatory network in HCC proliferation [103, 128, 129]. As to the cell cycle regulated by lncRNAs and its roles in HCC proliferation, Cao et al. reported that lncRNA UFC1 binds to mRNA stabilizing protein HuR to upregulate the expression of β-catenin and a series of checkpoint proteins including CDK4/6, c-Myc and cyclin D1, leading to G1/S phase transition and enhanced proliferation of HCC cell [54]. Besides, many other lncRNAs such as PVT1, Linc00974 and HEIH can also facilitate the cell cycle progression via interacting with NOP2, miR-642 and EZH2, respectively, thereby promoting the proliferation of HCC [66, 130, 131].

### Dysregulating cellular energetics

Cancer cells reprogram their metabolic pathways to provide energy and building blocks. These metabolic shifts include upregulation of aerobic glycolysis, glutaminolysis, lipogenesis and so on. To be specific, aerobic glycolysis means that cancer cells prefer to glycolysis instead of mitochondrial oxidative phosphorylation even in the presence of sufficient oxygen, which is also termed as “Warburg effect”, being the predominant way for energy production of cancer cells. Another metabolic shift glutaminolysis describes a biochemical reaction by which glutamine is lysed to yield a series of metabolites. This pathway is tightly related to the generation of NADPH and the proper function of cellular antioxidant machinery, facilitating cancer cell survival under oxidative stress. In addition, lipogenesis enhances membrane biosynthesis and energy storage, which is critical for cancer development [132]. Mechanistically, key metabolic enzymes usually undergo gain- or loss-of-function during those processes, which is frequently observed in most malignancies including HCC.

Particularly, lncRNA has been proposed as a critical factor in the dysregulation of metabolic enzymes and aberrant energetic mode of HCC cells, such as the upregulation of aerobic glycolysis, glutaminolysis, and lipogenesis (Fig. 4b). For instance, aerobic glycolysis can be upregulated by the activation of HK2 and the inactivation of AMPK, which have been addressed in the progression of HCC [133, 134]. Lin et al. have recently shown that lncRNA TUG1 is capable of stimulating the transcription of miR-455-3p and downregulates the protein level of AMPKβ2 by targeting its mRNA. This LncRNA-mediated AMPK silencing reactivates the expression of HK2, resulting in an elevated glycolytic capacity of HCC cells [58]. Besides, overexpression of GLS1, which catalyze the biogenesis of glutamate, making HCC cells addicted to glutaminolysis. LncRNA HOTTIP upregulates the expression of GLS1, thereby promoting the glutaminolysis and the proliferation of HCC cells. Inhibition of HOTTIP by endogenous miRNAs miR-192/204 downregulates the expression of GLS1 and is associated with a reduced level of glutaminolysis in HCC [67]. Another metabolic switch lies in the abnormal lipid metabolism pathway. LncRNA HULC modulates lipogenesis in HCC through activating ACSL1, the subunit of acyl-CoA synthetase that produces acyl-CoAs for the lipid synthesis. Mechanistically, HULC inhibits miR-9 to reactivate the expression of PPARA, which actively transcribes ACSL1 to accumulate intracellular cholesterol and HCC progression [64]. Another lncRNA H19 can also participate in lipogenesis by interacting with PTBP1, therefore maintaining the homeostasis of lipid metabolism in hepatocytes, and deletion of H19 is sufficient to abolish liver steatosis induced by high-fat-diet, suggesting its potential roles in HCC [135].

### Acquiring CSC properties

Stem cells preserve the abilities to replicate indefinitely and differentiate into specialized cell types, which are indispensable for the regeneration of damaged tissues. Liver holds remarkable regenerative capacity, where hepatic stem cells play key roles. However, cancer stem cells (CSCs) represent a major obstacle for cancer treatment and account for most cancer drug resistance and relapse. For HCC, several stemness markers such as CD133, CD24, EpCAM, ICAM-1 have been identified, and these HCC stem cells are believed to play critical roles in progression and chemoresistance of HCC [136, 137]. Importantly, multiple signalings such as Wnt-β-catenin, JAK-STAT, and Hippo-YAP pathways are involved in the maintenance of HCC stem cells. Briefly, activation of Wnt-β-catenin is associated with the expression of HCC stem cell markers such as CD133 and EpCAM, and reduced response to chemotherapeutic agents [138, 139]. Moreover, STAT3 is capable of simulating the expression of NANOG, a well-known pluripotency sustaining factor, thus promoting the expansion of CD24(+) HCC stem cells [140]. In addition, Hippo-YAP signaling controls the size of organism, the regeneration of liver, and the stemness of cells. Cai et al. reported that activation of Hippo-YAP can also increase the population of CD24(+) HCC stem cells, and is related to a more aggressive phenotype in HCC patients [141].

Accumulating evidence have suggested critical roles of lncRNAs in the regulation of Wnt-β-catenin, JAK-STAT, Hippo-YAP and other signaling pathways, affecting the stemness of HCC cells (Fig. 4c). Firstly, lncRNAs are able to regulate Wnt-β-catenin pathways in either post-transcriptional or post-translational level. For example, lncRNA DANCR binds to the mRNA of β-catenin to prevent its degradation by miRNAs, increasing the proportion of HCC cells with stemness features and elevated their tumor-seeding ability [61]. Another lncRNA lnc-β-Catm recruits EZH2 to promote the methylation of β-catenin at K49, inhibiting its ubiquitination and upregulates its stability, supporting the self-renewal of HCC stem cells [59]. Interestingly, lncTCF7 activates Wnt signaling and maintains HCC cell stemness by directly upregulating TCF7, which is probably not related with β-catenin [142]. Secondly, lncRNAs have been shown to either upregulate or downregulate the HCC cell stemness via promoting or suppressing IL-6-STAT signaling, respectively. Chen et al. have found that LncSox4 interacts with STAT3 and recruits it to the promoter of Sox4 to stimulate its transcription, conferring HCC cells with stem properties and results in HCC initiation [143]. In contrast, another lncRNA DILC functions as a tumor suppressor to inhibit the expansion of HCC stem cells via inhibiting IL6-STAT3 pathway [68]. Thirdly, Hippo signaling and its transcription cofactor YAP/TAZ are also implicated in liver CSCs. For instance, lncBRM interacts with the BRM protein to modulate the switch from BRM to BRG1 promoting the assembly of BRG1-embedded BAF complex, which activates YAP1 expression and supports self-renewal of HCC stem cells [144]. In addition, other lncRNAs such as PVT1 and ICR have been also shown to upregulate the stemness of HCC cells through binding with NOP2 protein and ICAM-1 mRNA, respectively, leading to HCC progression and predicting poor clinical outcomes [66, 69].

### Activating invasion and metastasis

Tumor metastasis causes the great majority of cancer-related death. Briefly, metastasis begins with epithelial-mesenchymal transition (EMT) program where epithelial marks (E-cadherin, ZO-1, etc.) are downregulated and mesenchymal marks (vimentin, ZEB1, etc.) are overexpressed within cells. Then mesenchymal-like cells invade into vessels where some of them become anoikis resistant and translocate to distant organ to form metastases. Particularly, the metastasis of HCC shares considerable similarities with other cancer types, whereas EMT program and enhanced angiogenesis have been addressed as critical events driving HCC metastasis [145, 146].

Several lncRNAs are capable of regulating the expression of certain EMT markers and the formation of additional blood vessels, affecting HCC metastasis (Fig. 4d). For example, two lncRNAs ATB and ZFAS1 are capable of sponging miR-200 and miR-150, respectively. It allows for the reactivation of ZEB1, a mesenchymal mark that inhibits E-cad expression, thus promoting the metastasis of HCC [40, 70]. Besides miRNA sponge, lncRNAs can also directly regulate the expression of ZEB1 without involving miRNAs. One of such cases is that lncRNA ZEB1-AS1 stimulates the expression of ZEB1 by elevating its promoter activity thus promoting the metastasis of HCC, and patients with ZEB1-AS1 hypomethylation are associated with high metastatic recurrence and poor survival [147]. Moreover, another key mesenchymal mark vimentin can interact with lncRNA Dreh, which functions as a tumor suppressor in HCC. This binding disrupts the cytoskeleton structure, leading to the suppression of HCC metastasis [24]. As to the roles of lncRNA-mediated regulation of angiogenesis during HCC metastasis, Yu et al. reported that lncRNA MVIH represses the secretion of PGK1, an inhibitory regulator of angiogenesis, therefore promoting the vessel formation and HCC metastasis [71]. Importantly, it has been widely accepted that hypoxia largely contributes to tumor angiogenesis, at least partly due to the overexpression of HIF proteins. This process involves lncRNA LET, which has been found to downregulate the expression of HIF-1α thereby suppressing hypoxia-mediated HCC metastasis [44]. It is worth mentioning that many other lncRNAs, including FTX, MUF, miR503HG, NEF, TSLNC8, etc. are capable of regulating HCC metastasis through distinct mechanisms, which have been described elsewhere [101, 102, 148–150].

## Diagnostic and therapeutic potentials of lncRNA in HCC

To date, the diagnosis of HCC largely relies on ultrasonography imaging and AFP measurement. Indeed, high-risk populations are recommended to perform ultrasonography screening, and those patients who received more frequent imaging have been associated with better survival [151]. However, this surveillance imaging is inadequate to visualize early stage HCC with only 47% sensitivity [152]. Besides, the widely used HCC biomarker AFP has 52.9% sensitivity and 93.3% specificity, which can be further improved when combined with ultrasonography imaging [153]. However, other factors such as HCV infection has been shown to elevate AFP level in the absence of HCC [154]. Furthermore, a recent study even suggested that neither ultrasonography imaging nor AFP measurement decreases the mortality of HCC patients [155]. Apart from lacking reliable biomarkers, limited therapeutic options also contribute to HCC-related death. Currently, surgical methods such as resection and liver transplantation are the only possible curative approaches for early HCC, while HCC in late stage is largely incurable. Therefore, novel biomarkers and therapeutic targets are urgently needed for improving the diagnosis and treatment of HCC.

### LncRNAs are potential biomarkers for HCC diagnosis

As previously discussed, a large number of lncRNAs are aberrantly expressed in HCC compared with normal liver tissue, which is useful to distinguish HCC patients from healthy cohorts. However, some of those lncRNAs are also shown aberrant expression patterns in other cancer types or non-cancerous situations such as cirrhosis or liver injury, resulting in reduced reliability. Thus, lncRNAs combined with other molecules, especially known HCC biomarker AFP, is more likely to be a desirable HCC diagnosis method instead of evaluating lncRNAs alone. For instance, the combination of two lncRNAs UCA1 and WRAP53 with AFP achieves sensitivity up to 100% [20]. Similarly, the combination of another two lncRNAs PVT1 and uc002mbe.2 with AFP have been also shown to perform much better than AFP alone in HCC diagnosis [156]. Besides AFP, other molecules including miRNAs or mRNAs can also predict HCC in combination with lncRNAs, which have been reviewed elsewhere [157]. In addition to their diagnostic value, various lncRNAs might also be potential prognostic markers in HCC. For instance, aforementioned lncRNA WRAP53 is also an independent prognostic biomarker to predict high relapse rate of HCC patients [20]. Not surprisingly, lncRNAs involved in tumor metastasis might well hold prognostic value. For example, lncRNA-ATB, which is upregulated in HCC metastases, predicts poor prognosis of HCC patients [40]. In contrast, lncRNA miR503HG with metastasis-suppressing function is a favorable prognostic indicator [148].

Compared with sampling solid tumor tissues, liquid biopsy is largely non-invasive thus being an ideal diagnostic approach. Importantly, several HCC-related lncRNAs can be present in the body fluid (circulating lncRNAs), as is the case with aforementioned lncRNAs UCA1, WRAP53, PVT1 and uc002mbe.2. Also, the representative HCC-promoting lncRNA HULC is detectable in blood sample and can be easily quantified by conventional real-time qPCR [17]. These findings have suggested a noninvasive approach for HCC diagnosis through circulating lncRNA measurement. Further studies have revealed that these circulating lncRNAs origin from the secretion of exosomes or other extracellular vesicles (EVs) such as microvesicles and apoptotic bodies, which protect them from RNase-mediated degradation in body fluid [158, 159]. One of such examples is linc-RoR, a hypoxia-responsive lncRNA that released from HCC cells by EVs [43]. This EV-mediated lncRNA transferring contributes to rapid cell-to-cell communication and intercellular signaling transduction. Furthermore, these EV-engulfed lncRNAs can be isolated from body fluid by various methods such as ultracentrifugation and ultrafiltration, providing efficient tools to analyze circulating lncRNAs that possibly serve as HCC biomarkers [160].

### LncRNAs as promising HCC therapeutic targets

To date, the prognosis of HCC is overall poor, which is at least in part due to lack of therapeutic target. The most used targeted drug in HCC is sorafenib that targeting RTKs, however, sorafenib resistance is frequently observed during HCC treatment [161]. Thus, novel target is required for improving the prognosis of HCC patients. The critical roles of lncRNAs in HCC make them promising drug targets for novel therapeutic interventions. Furthermore, lncRNA-targeting approaches hold some advantages over protein-targeting methods in terms of base-pairing principle is much more straightforward than designing a specific protein-binding inhibitor. These lncRNA-targeting approaches mainly include antisense oligonucleotides (ASOs) and RNA interference (RNAi), both of which have been shown favorable anticancer activities against HCC via targeting lncRNAs. For instance, ASO-mediated linc00210 silence has been shown to repress the self-renewal and invasion of HCC cells, and knockdown of lncRNA CASC9 by RNAi significantly reduced the tumor formation in a HCC mouse model [72, 162]. Briefly, ASOs are short single-stranded DNAs that bind to target lncRNA to form DNA-RNA complex, which can be recognized and cleaved by RNase H. In contrast, RNAi are short double-stranded RNAs that have to be loaded into AGO2 protein to form an RNA-induced silencing complex (RISC), then interact with target lncRNA to yield RNA-RNA complex and mediate lncRNA silence [163]. In view of these differences, ASOs and RNAi probably display distinct silencing efficiency upon various factors, such as the subcellular location of target lncRNAs. Lennox et al. reported that ASOs function better than RNAi in nuclei, whereas RNAi is more effective than ASOs for targeting cytoplasmic lncRNAs [164]. It can be at least partially due to the fact that RNase H is mainly present in nuclei while RISC predominantly function in the cytoplasm [92, 165]. Besides knockdown of oncogenic lncRNAs, delivery of tumor suppressive lncRNAs can be an alternative strategy. For instance, lncRNA PRAL serves as a tumor suppressor through stabilizing p53, and gene delivery of PRAL through adenovirus vector has been shown to significantly inhibit HCC growth in tumor-bearing mice, suggesting its potential clinic value for HCC treatment [73]. It is worth noted that the current pipeline of HCC-targeted ASOs and RNAi is broad, for both of which have been already applied in the treatment of HBV. Briefly, ASOs and siRNAs are modified by chemical conjugates (such as N-acetylgalactosamine, GalNAc) [166] or formulated in delivery vehicles (such as lipid nanoparticles) [167], thereby achieving an optimized pharmacokinetic profile. These commercial experiences in HBV treatment using ASOs and RNAi provide a substantial basis for lncRNA interference and HCC therapy.

## Conclusions

Cancer relevant lncRNAs are gradually becoming one of the hottest issues in the RNA biology and oncology. Current evidences reveal that aberrant transcription or processing events can lead to the upregulation of oncogenic lncRNAs or silence of tumor suppressive lncRNAs, which bind to DNA, RNA or proteins. As a result, lncRNAs regulate the expression, localization, stability, activity and other properties of their binding partners, raising a series of cancerous phenotypes such as persistent proliferation, abnormal metabolism, elevated stemness and metastasis, leading to tumorigenesis and progression of HCC. In terms of their crucial roles, a part of lncRNAs that present in body fluid hold value of serving as potential HCC biomarkers alone with high sensitivity, or in combination with other molecules to improve specificity. Thus, modulation of lncRNA expression can be a novel therapeutic strategy for the treatment of HCC.

Though remarkable progress has been achieved in this field, the functions of most lncRNAs are still elusive. The lack of functional studies sometimes challenges the importance of lncRNAs, which can be partially due to their overall less sequence conservation compared with protein-coding genes [168]. Nevertheless, lncRNAs tend to maintain evolutionarily conserved secondary structure in the present of considerable sequence variation [169]. Moreover, it has been proposed that selective pressure may act on lncRNAs at the secondary structure to constrain sequence variation, which depletes mutations in structural regions with low fitness [170]. These findings suggest that sequence polymorphism may not be selected if it does not induce significant structural alterations, resulting in a lack of sequence conservation of lncRNAs. In contrast, sequence conservation of protein-coding genes is much higher, for the insert or deletion of even a single nucleotide is sufficient to result in frameshift and complete loss of function. Thus, for lncRNAs, secondary structure might serve as not only evolutionary constraint, but also the main functional unit to participate in biological processes, including HCC progression. For instance, the three stem-loop structure in lncRNA PRAL is required for its function as the molecular scaffold and tumor suppressor against HCC [73]. Together, these findings suggest that the function of lncRNAs might also be largely determined by their secondary structure in addition to the primary sequence. However, the secondary structure of lncRNAs seems to receive no enough interest, not to mention revealing the connection between lncRNA secondary structure and their biological functions. In contrast, RNA modifications, especially N6-methyladenosine (m6A) has received more attention and become a new research hotspot in the functional study of lncRNA. The well-known lncRNA Xist is one of such lncRNAs undergo m6A modification, which is critical for its function to mediate transcriptional repression [171]. Mechanistic studies revealed that m6A frequently regulates the degradation of modified lncRNAs, including those related with HCC. For example, m6A modification upregulates the stability of LINC00958, leading to its overexpression in HCC and promotes cell proliferation and invasion [172]. This effect may result from an enhanced binding of lncRNA with protective partner, or a disassociation with RNA decay machinery. In fact, m6A modification results in structural changes on lncRNAs, which regulate the interaction between them and their binding partners [173]. As mention above that lncRNAs largely function through their binding partners, the regulation of lncRNAs by m6A modification should be comprehensive and ubiquitous.

Another concern is the lack of lncRNA knockout animal model, which can be attributed to many reasons. As previously discussed, lncRNAs are impervious to sequence mutations, which means that certain genetic perturbations used to knockout protein-coding genes may not function well when applied to knockout lncRNAs. Besides, many lncRNAs have overlap with protein-coding genes, making it difficult to specifically knockout a lncRNA without affecting overlapping genes. As a result, knockdown of lncRNAs at the transcription level (e.g. ASOs and RNAi as described above) has been used as an alternative strategy to investigate their functions in vivo. However, this knockdown method may not be sufficient to eliminate lncRNAs compared with a knockout approach, and the introduction of exogenous molecules may be toxic to organisms and induce off-target effects [174]. Therefore, lncRNA knockout mouse models are more reliable and necessary for revealing their functions, whereas no such models have been used in the studies of HCC so far. Notably, some possible lncRNA knockout methods have been proposed including the deletion of lncRNA genes completely as the conventional strategy, deletion of lncRNA promoter to prevent transcription, and insert of polyadenylation signal to terminate the transcription at the outset [175]. Moreover, lncRNA knockout mice have been used in other cancer types such as melanoma and noncancerous biological processes such as brain development [176, 177]. Therefore, this model will be certainly used to facilitate the investigation of HCC in the near future.

## Data Availability

All the data obtained and/or analyzed during the current study were available from the corresponding authors on reasonable request.

## Abbreviations

lncRNA: long noncoding RNA
HCC: Hepatocellular carcinoma
HBV: Hepatitis B virus
HCV: Hepatitis C virus
NAFLD: Non-alcoholic fatty liver disease
AFP: A-fetoprotein
ncRNA: noncoding RNA
siRNA: small interfering RNA
miRNA: microRNA
piRNA: PIWI-interacting RNA
ROS: Reactive oxygen species
XIST: X-inactive-specific transcript
HULC: Hepatocellular carcinoma up-regulated long non-coding RNA
MALAT1: Metastasis-associated lung adenocarcinoma transcript 1
MENβ: Multiple endocrine neoplasia β
MEG3: Maternally expressed gene 3
PXN-AS1: PXN antisense transcript 1
circRNA: circular RNA
TUG1: Taurine-upregulated gene 1
RUNXOR: RUNX1 overlapping RNA
TINCR: Terminal differentiation-induced ncRNA
THOR: Testis-associated highly conserved oncogenic long non-coding RNA
Mhrt: Myosin heavy-chain-associated RNA transcripts
ANRIL: CDKN2B antisense RNA 1
lnc-β-Catm: lncRNA for β-catenin methylation
GIHCG: Gradually increased during hepatocarcinogenesis
UCA1: Urothelial cancer associated 1
UFC1: Ubiquitin-fold modifier conjugating enzyme 1
DANCR: Anti-differentiation ncRNA
CASC9: Cancer Susceptibility 9
RTK: Receptor tyrosine kinase
HOTTIP: HOXA distal transcript antisense RNA
CSC: Cancer stem cell
DILC: Downregulated in liver cancer stem cells
PVT1: Plasmacytoma variant translocation 1
ATB: lncRNA-activated by TGF-β
ZFAS1: ZNFX1 antisense RNA 1
Dreh: lncRNA down-regulated expression by HBx
MVIH: lncRNA associated with microvascular invasion in HCC
PSTAR: p53-stabilizing and activating RNA
HDGF: Hepatoma-derived growth factor
WRAP53: WD repeat containing, antisense to TP53
IFI6: Interferon Alpha-Inducible Protein 6
CMPK2: CMP kinase 2
EGOT: Eosinophil granule ontogeny transcript
LINE1: Long interspersed nuclear elements 1
LALR1: Long noncoding RNAs associated with liver regeneration 1
DSCR8: Down syndrome critical region 8
SNHG5: Small nucleolar RNA host gene 5
NEAT1: Nuclear-enriched abundant transcript 1
RoR: Regulator of reprogramming
LET: Low Expression in Tumor
Khps1: Antisense orientation to the proto-oncogene SPHK1
UPAT: UHRF1 Protein Associated Transcript
HOTAIR: HOX Antisense Intergenic RNA
FTX: An XIST regulator transcribed from the X chromosome inactivation center
MUF: MSC-upregulated factor
FAL1: Focally amplified lncRNA on chromosome 1
lncKdm2b: a divergent lncRNA for Kdm2b gene
NORAD: Noncoding RNA activated by DNA damage
PRAL: p53 regulation-associated lncRNA
ENOD40: Early nodulin 40
NCBP2-AS2: NCBP2-antisense 2
HEIH: Long noncoding RNA high expression in hepatocellular carcinoma
ICR: ICAM-1-Related Noncoding RNA
ZEB1-AS1: ZEB1 antisense 1
miR503HG: the host gene of miR503
NEF: TSLNC8, tumor suppressor long noncoding RNA on chromosome 8p12
